# Electrostatic model of dielectric elastomer generator based on finite element

**DOI:** 10.1038/s41598-021-93486-0

**Published:** 2021-07-20

**Authors:** Jianbo Cao, Gangqiang Lu, E. Shiju, Zhao Gao, Tianfeng Zhao, Wenjun Xia

**Affiliations:** 1 College of Information Science and Engineering, Jiaxing University, Jiaxing, 314001 Zhejiang People’s Republic of China; 2 College of Engineering, Zhejiang Normal University, Jinhua, 321004 Zhejiang People’s Republic of China; 3 Library, Jiaxing University, Jiaxing, 314001 Zhejiang People’s Republic of China

**Keywords:** Materials science, Physics

## Abstract

When dielectric elastomer materials are used for power generation, bias voltage is applied at both ends of dielectric elastomer film, and there are equal amounts of heterogeneous charges on both sides of the film, so Maxwell electrostatic force is always coupled in the process of power generation. In order to investigate the distribution of Maxwell stress in dielectric elastomer material under electric field, the electrostatic model of dielectric elastomer generator is established in COMSOL finite element simulation software environment in this paper. The distribution of electrostatic force is studied from two aspects of theoretical derivation and simulation, and the magnitude and direction of electrostatic force are determined. The simulation results show that the Maxwell electrostatic force can be equivalent to the tensile force along the film plane and the extrusion force perpendicular to the plane, and they are the same.

## Introduction

Dielectric elastomer (DE) is a new type of smart material that could be used as an actuator to convert electrical energy into mechanical energy. In recent years, it has been studied in the field of power generation for energy recovery^1–4^. DE has some advantages, such as large strain, light weight and high flexibility^5–8^. Compared with other power generation materials, DE has higher energy conversion efficiency and specific energy density^9,10^. Therefore, DE has good application prospects and application fields, such as wind generators, wave energy generators, body-worn generators, etc.^11,12^.

Dielectric Elastomer Generator (DEG) refers to a “sandwich” three-layer variable capacitor that uses a dielectric elastomer as a carrier, two layers of upper and lower flexible electrodes, and an electrode wire. Its power generation principle is variable capacitor power generation^13^. Under the action of external mechanical force, the capacitance of DE increases when DE is stretched. At this time, under the action of bias power supply, the initial charge is increased. When DE shrinks, the anisotropic charges in the upper and lower surface electrodes are pushed away due to the increase of thickness, and the isotropic charges are squeezed closer due to the decrease of area, thus increasing the charge voltage. That is, the capacitance decreases, the charge remains unchanged, and the voltage increases, so that the mechanical energy can be converted into electrical energy^14^.

Dielectric elastomer, as an intelligent energy exchange material, can be used not only in bionic driving materials, but also in the field of power generation. When used in bionic driving materials, Maxwell electrostatic force is the essential driving source of driving materials. Applying voltage to the upper and lower electrodes of the film, the upper and lower electrodes accumulate equal charges with opposite polarities, which results in Maxwell electrostatic force driving film deformation. When applied in the field of power generation, bias voltage is applied at both ends of the film, and the same amount of heterogeneous charges are accumulated on both sides of the film, so Maxwell electrostatic force is always coupled in the process of power generation, which affects the electromechanical characteristics of DEG^15–18^.

In order to investigate the distribution of Maxwell stress in dielectric elastomer materials under electric field during power generation^19,20^, the electrostatic model of dielectric elastomer materials was established in COMSOL finite element simulation software environment based on theoretical deduction. Based on the simulation results, the electromechanical characteristics of DEG are studied.

## Theoretical Model

Electromagnetic field essentially generates volume force in the medium. The resultant force of electromagnetic field acting on the medium can be calculated by the method of volume force density, or by the surface force produced by Maxwell stress tensor^21^. The Maxwell stress tensor can be expressed as:

1 $$ {\mathbf{T = ED}}^{{\mathbf{T}}}  - \frac{{\mathbf{1}}}{{\mathbf{2}}}{\mathbf{(E}} \cdot {\mathbf{D)I}} $$

where ${\mathbf{E}}$ is the electric field strength and ${\mathbf{D}}$ is the potential shift. And ${\mathbf{E}}$ is the gradient of voltage, which can be expressed as:

2 $$ {\mathbf{E = }} - \nabla {\mathbf{V}} $$

where ${\mathbf{V}}$ is the voltage. Potential shift ${\mathbf{D}}$ can be expressed by electric field strength ${\mathbf{E}}$.

3 $$ {\mathbf{D}} = \varepsilon _{0} \varepsilon _{{\text{r}}} {\mathbf{E}} $$

where $\varepsilon _{0}$ is the dielectric constant in vacuum and $\varepsilon _{r}$ is the relative dielectric constant.

Thus, formula (1) can be simplified as follows:

4 $$ {\mathbf{T = }}\varepsilon _{0} \varepsilon _{r} {\mathbf{EE}} - \frac{{\mathbf{1}}}{{\mathbf{2}}}\varepsilon _{0} \varepsilon _{r} {\mathbf{E}}^{2} {\mathbf{I}} $$

**Figure 1:**
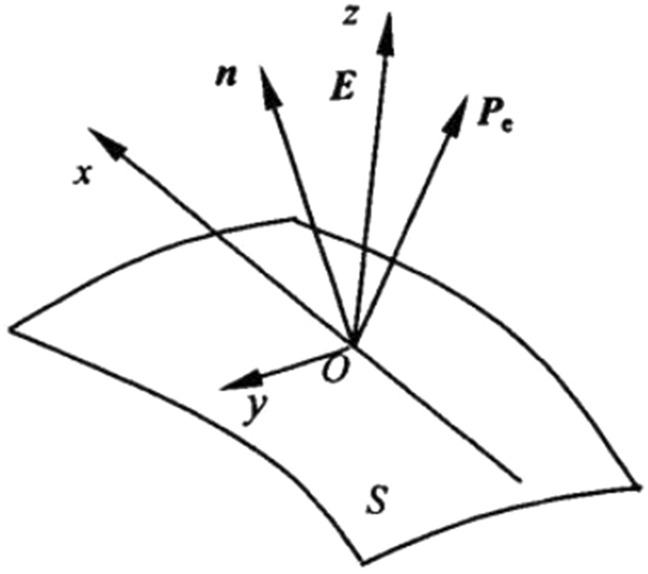
Surface force caused by Maxwell stress.

The surface force produced by Maxwell stress tensor in DE film under electric field is given below, as shown in Fig. 1. Let O be a point on a surface of thin film and establish coordinate system XYZ through O point^22^. The normal unit vector of O-crossing point is ${\mathbf{n}}$, and the electric field ${\mathbf{E}}$ and Z axes are in the same direction. In this coordinate system, the three components of electric field ${\mathbf{E}}$ are:

5 $$ {\mathbf{E}}_{{\mathbf{x}}} {\mathbf{ = 0,E}}_{{\mathbf{y}}} {\mathbf{ = 0,E}}_{{\mathbf{z}}} {\mathbf{ = E}} $$

Then Maxwell stress tensor can be expressed in matrix form:

6 $$ {\mathbf{T}} = \left[ {\begin{array}{*{20}c}    { - \frac{{\varepsilon _{0} \varepsilon _{r} {\mathbf{E}}^{2} }}{2}} & 0 & 0  \\    0 & { - \frac{{\varepsilon _{0} \varepsilon _{r} {\mathbf{E}}^{2} }}{2}} & 0  \\    0 & 0 & {\frac{{\varepsilon _{0} \varepsilon _{r} {\mathbf{E}}^{2} }}{2}}  \\   \end{array} } \right] $$

The electric field force acting on the unit area of the film is as follows:

7 $$ {\mathbf{P}}_{e} {\mathbf{ = T}} \cdot {\mathbf{n}} $$

If ${\mathbf{n}}$ and X, Y and Z axes are in the same direction in turn, the electric field force components in the three main directions can be obtained as follows:

8 $$ {\mathbf{P}}_{{{\text{ex}}}} {\mathbf{ = }} - \frac{{\varepsilon _{0} \varepsilon _{r} {\mathbf{E}}^{2} }}{2},\quad {\mathbf{P}}_{{{\text{ey}}}} {\mathbf{ = }} - \frac{{\varepsilon _{0} \varepsilon _{r} {\mathbf{E}}^{2} }}{2},\quad {\mathbf{P}}_{{{\text{ez}}}} {\mathbf{ = }}\frac{{\varepsilon _{0} \varepsilon _{r} {\mathbf{E}}^{2} }}{2} $$

where *P*_ex_, *P*_ey_ and *P*_ez_ components are the projections of *P*_e_ along the x-axis, y-axis, and z-axis, respectively. According to the projection result, *P*_ex_ component and *P*_ey_ component are positive, and *P*_ez_ component is negative.

Next, from the point of view of energy conservation, the force acting on DE film under electric field is analyzed.

The DE film coated with electrodes is analogized to a deformable parallel plate capacitor. Therefore, when the capacitor is charged, an equal amount of dissimilar charge Q is accumulated at both ends of the positive and negative electrodes. At this time, the capacitance of the film can be expressed as:

9 $$ C = \frac{Q}{U} = \varepsilon _{0} \varepsilon _{{\text{r}}} \frac{S}{{\text{d}}} $$

Among them, *U* denotes the voltage at both ends of the capacitor, *S* denotes the area of the capacitor plates, and *d* denotes the distance between the capacitor plates.

According to the formula of capacitive electric energy: $W = \frac{1}{2}CU^{2}$, when the external power supply charges the DE film, the electric energy is:

10 $$ W_{e}  = \frac{{Q^{2} }}{{2C}} = \frac{{Q^{2} d}}{{2\varepsilon _{0} \varepsilon _{{\text{r}}} S}} $$

After applying voltage, the thickness and area of the film are deformed due to the existence of Maxwell electrostatic force, so the increment of $W_{e}$ is as follows:

11 $$ \delta W_{e}  = \frac{Q}{C}\delta Q + W_{e} \left( {\frac{1}{d}\delta d - \frac{1}{S}\delta S} \right) $$

The first term in the formula represents the energy change caused by the change of charge when the power supply charges the thin film, and the second term represents the energy differential which is converted into mechanical energy during the deformation of the thin film. In the charging process, the work done by charge and external force is equal to the change of electric energy on DE film.

12 $$ \delta W_{e}  = \frac{Q}{C}\delta Q + \sigma _{{EA}} d\delta S + \sigma _{{EZ}} S\delta d $$

where $\sigma _{{EA}}$ is the equivalent stress of Maxwell stress in the plane direction and $\sigma _{{EZ}}$ is the equivalent stress of Maxwell stress in the thickness direction.

Comparisons (11) and (12) are available:

13 $$ W_{e} (\frac{1}{d}\delta d - \frac{1}{S}\delta S) = \sigma _{{EA}} d\delta S + \sigma _{{EZ}} S\delta d $$

It can be simplified as follows:

14 $$ \sigma _{{EA}}  =  - \frac{1}{2}\varepsilon _{0} \varepsilon _{{\text{r}}} \left( {\frac{U}{d}} \right)^{2}  =  - \frac{1}{2}\varepsilon _{0} \varepsilon _{{\text{r}}} E^{2} ,\quad \sigma _{{EZ}}  = \frac{1}{2}\varepsilon _{0} \varepsilon _{{\text{r}}} \left( {\frac{U}{d}} \right)^{2}  = \frac{1}{2}\varepsilon _{0} \varepsilon _{{\text{r}}} E^{2} $$

The above two methods show that the Maxwell stress has $\sigma _{{EA}}$ in the plane direction and $\sigma _{{EZ}}$ in the thickness direction, and both of them are equal in size and are $\frac{1}{2}\varepsilon _{0} \varepsilon _{{\text{r}}} E^{2}$.

## Simulation Study

**Figure 2:**
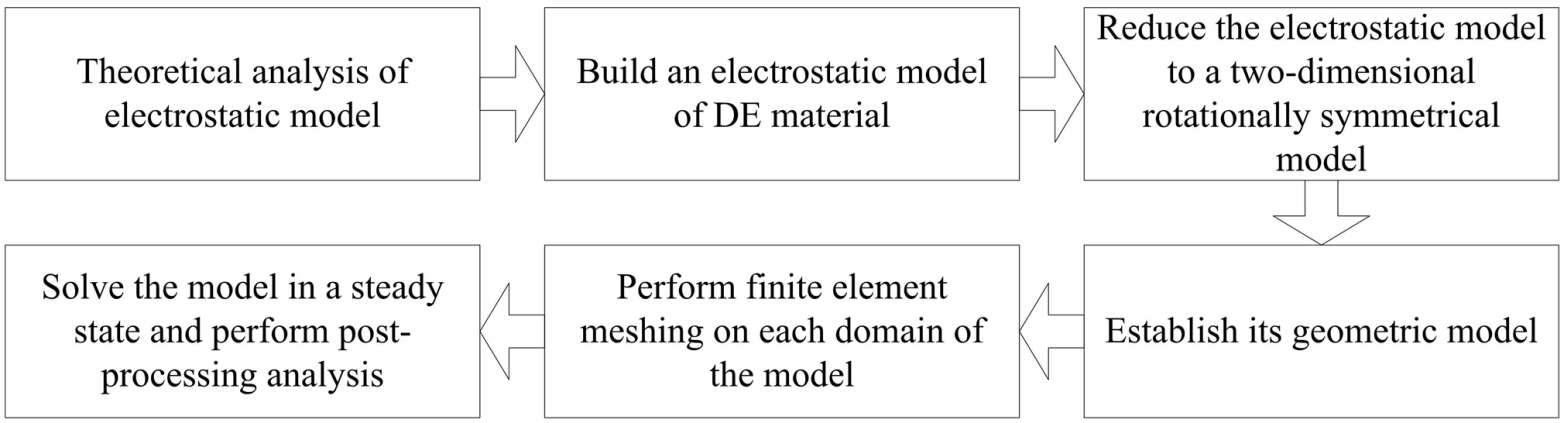
Flow chart for numerical simulation process of DEG electrostatic model based on finite element.

The flow chart for numerical simulation process of DEG electrostatic model based on finite element is shown in Fig. 2.

DE material will undergo stress and deformation under electric field, and there is a coupling between force field and electric field. From the above theoretical analysis, it is known that the Maxwell stress is generated when the electric field is applied. COMSOL Multiphysics finite element software is famous for its strong multi-physical field coupling characteristics. It can easily couple various physical fields and analyze their coupling characteristics^23^. COMSOL Multiphysics 5.0 was used in the study. In the electrostatic module of COMSOL Multiphysics, the electric field and force field are coupled based on Maxwell stress tensor. The formulas are as follows:

15 $$ {\mathbf{F = }}\nabla  \cdot {\mathbf{T}} $$

16 $$ {\mathbf{T = ED}}^{{\mathbf{T}}}  - \frac{{\mathbf{1}}}{{\mathbf{2}}}{\mathbf{(E}} \cdot {\mathbf{D)I}} $$

**Figure 3:**
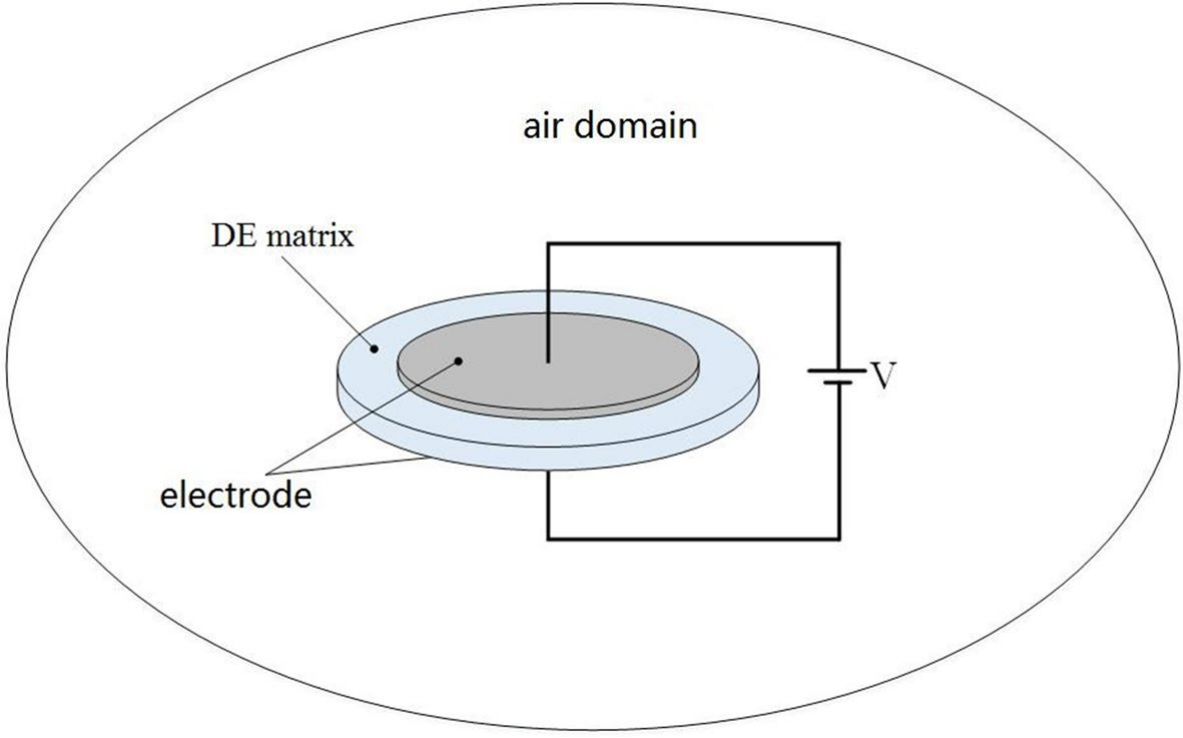
Schematic diagram of electrostatic model simulation.

In order to study the Maxwell stress distribution of DE material under electric field, an electrostatic model of DE material was established in COMSOL environment. In order to study the convenience, a rotationally symmetrical circular thin film sample is selected for analysis. The schematic diagram is shown in Fig. 3. The electrodes of the same size are coated on the upper and lower sides of the circular DE matrix. The DC voltage is applied to the upper and lower electrodes. The whole material is in an infinite air region, thus forming the whole electrostatic model.

**Figure 4:**
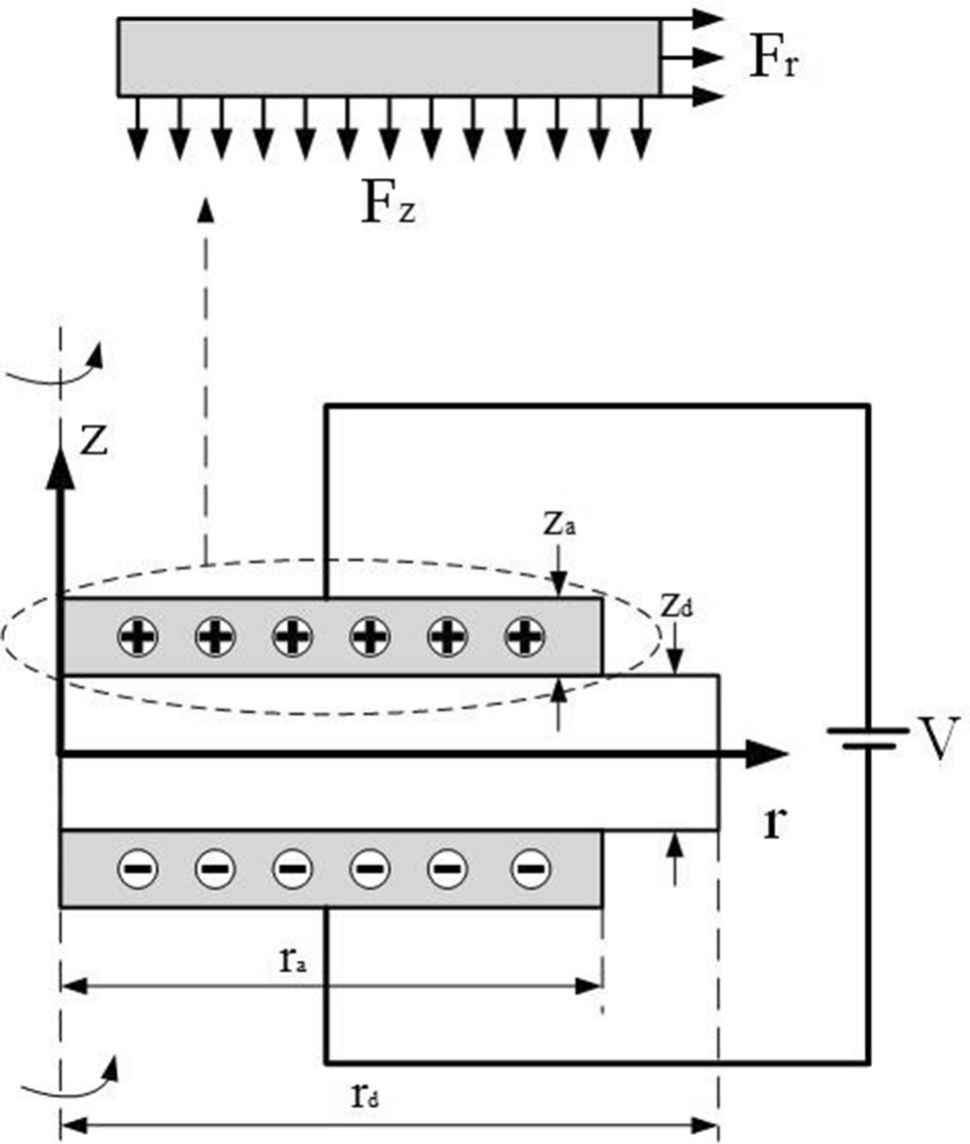
Symmetric rotation diagram of electrostatic model.

As we know from the above figure, the whole model is rotationally axisymmetric. In the finite element modeling, the electrostatic model can be reduced to a two-dimensional rotationally symmetric model for easy solution and analysis. The schematic diagram is shown in Fig. 4. As can be seen from the figure, after applying voltage, the upper and lower electrodes accumulate the same amount of heterogeneous charges. The heterogeneous charges gathered at the upper and lower electrodes attract each other and produce force $F_{z}$, while the same charges on the same side of the electrode repel each other and produce force $F_{r}$. Among them, $r_{d}$ and $z_{d}$ are the radius and thickness of DE matrix, $r_{a}$ and $z_{a}$ are the radius and thickness of coated electrode respectively, and the bias voltage is *V*.

**Figure 5:**
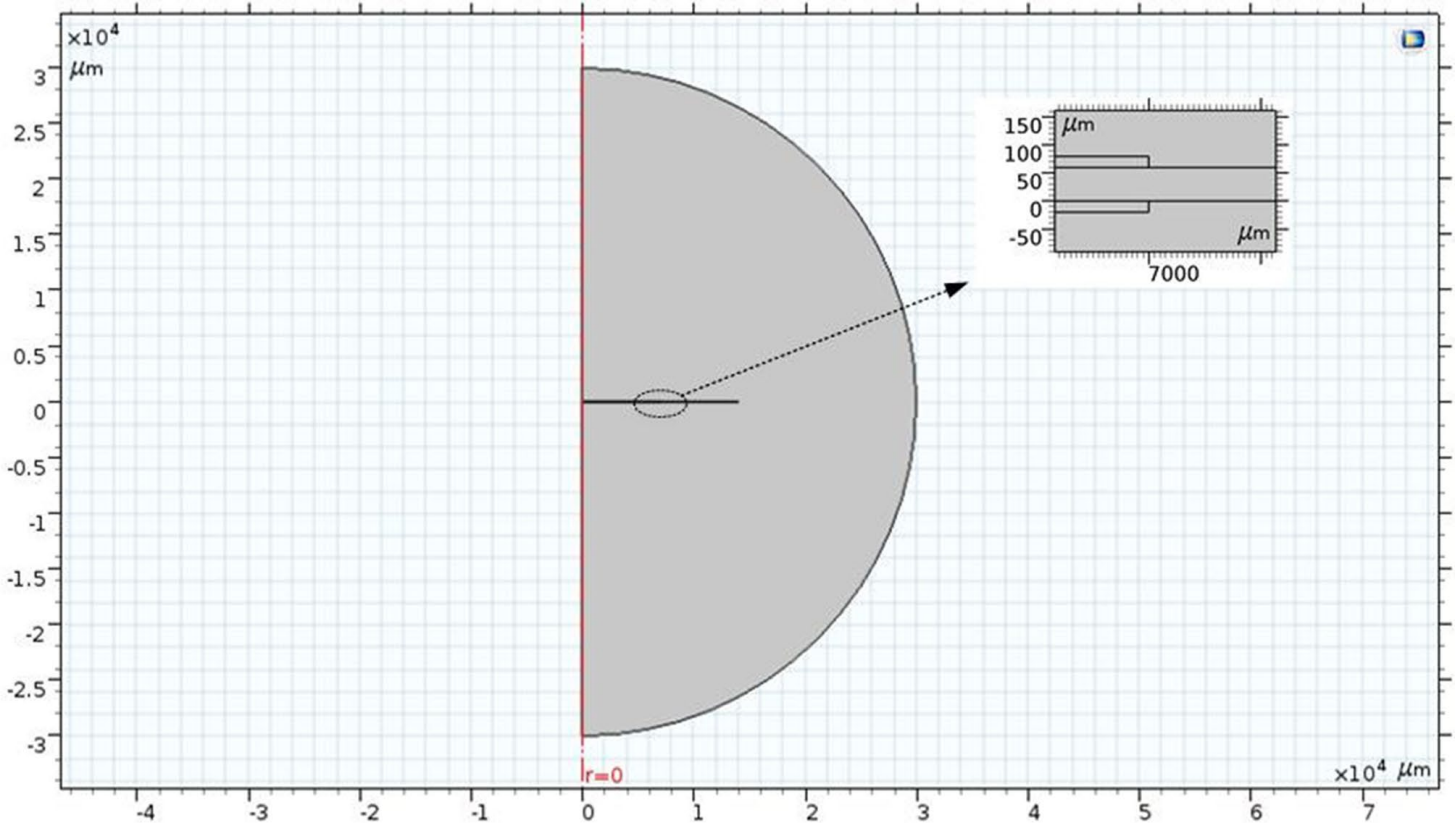
Geometric modeling diagram of electrostatic model simulation by COMSOL Multiphysics 5.0.

The electrostatic module of COMSOL Multiphysics is selected for two-dimensional rotational symmetry modeling. Firstly, the geometric model is established. The radius $r_{d}$ of DE matrix is 14 mm, the thickness $z_{d}$ of DE is 60 um, the radius $r_{a}$ of electrode is 7 mm, and the thickness $z_{a}$ is 20 um. All materials are contained in the circular air region, as shown in Fig. 5. The relative dielectric constant of DE film is 4.7 and that of air is 1. Physical field constraints are applied to the model, 3000 V DC voltage is added to the upper electrode, and the lower electrode is grounded to form potential difference. The application of voltage causes the charge to accumulate on the surface of the upper and lower electrodes, and the surface charge density is $\rho$. The divergence of the electric field strength is equal to the charge density divided by the dielectric constant. Substituting charge density = dielectric permittivity of medium × electric field strength. So Maxwell stress tensor formula can be expressed by charge density as follows:

17 $$ T = \frac{1}{2}\varepsilon _{0} \varepsilon _{{\text{r}}} E^{2}  = \frac{{\rho _{s} ^{2} }}{{2\varepsilon _{0} \varepsilon _{r} }} $$

where $\rho _{s}$ is the charge density.

**Figure 6:**
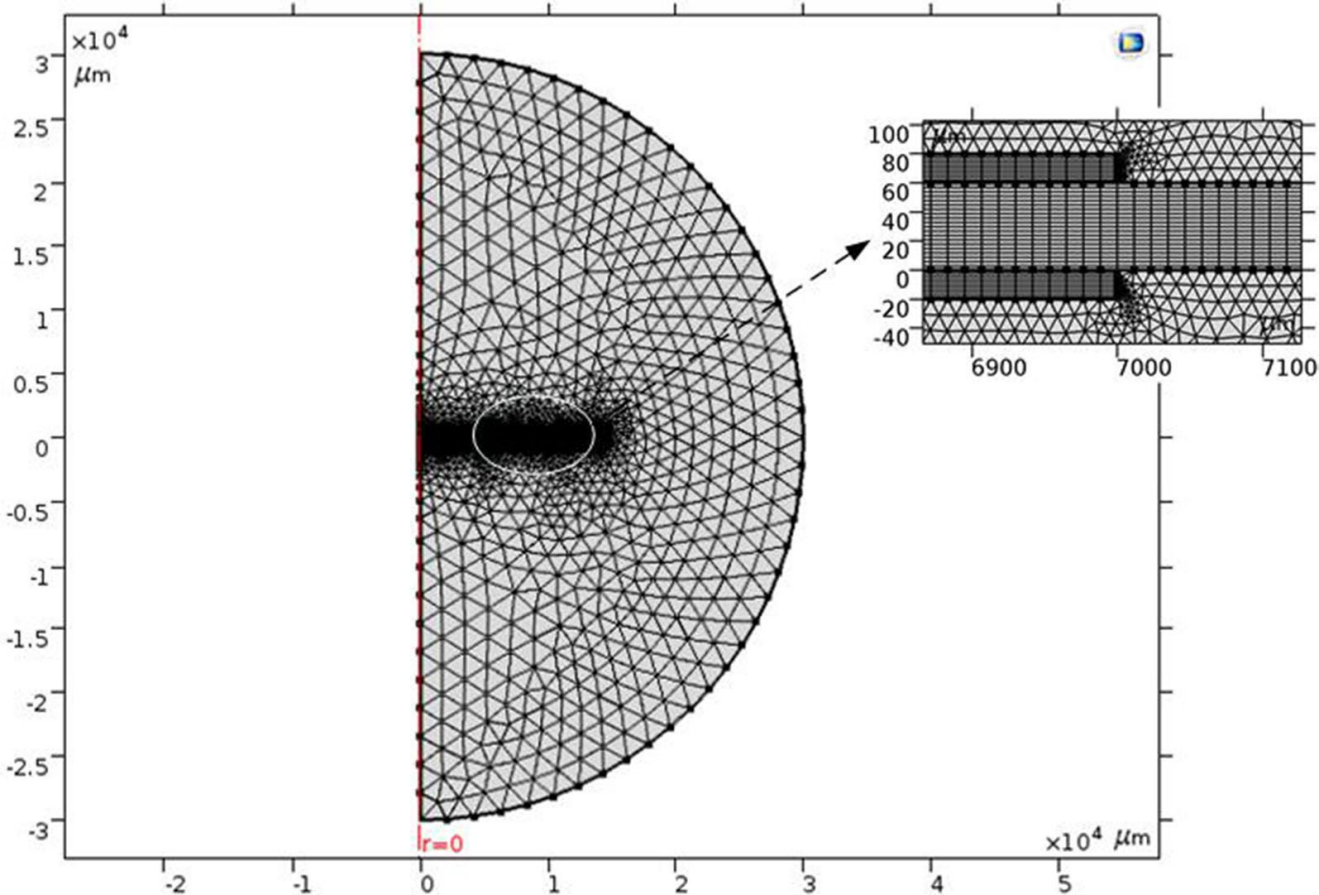
Grid generation of electrostatic model by COMSOL Multiphysics 5.0.

In order to improve the simulation accuracy, the DE matrix and electrodes are divided into regular quadrilateral meshes, and the boundary is refined. The triangular meshes are used in the external air domain, as shown in Fig. 6.

The steady-state solution of the model is carried out and the post-processing analysis is carried out.

## Results Analysis

**Figure 7:**
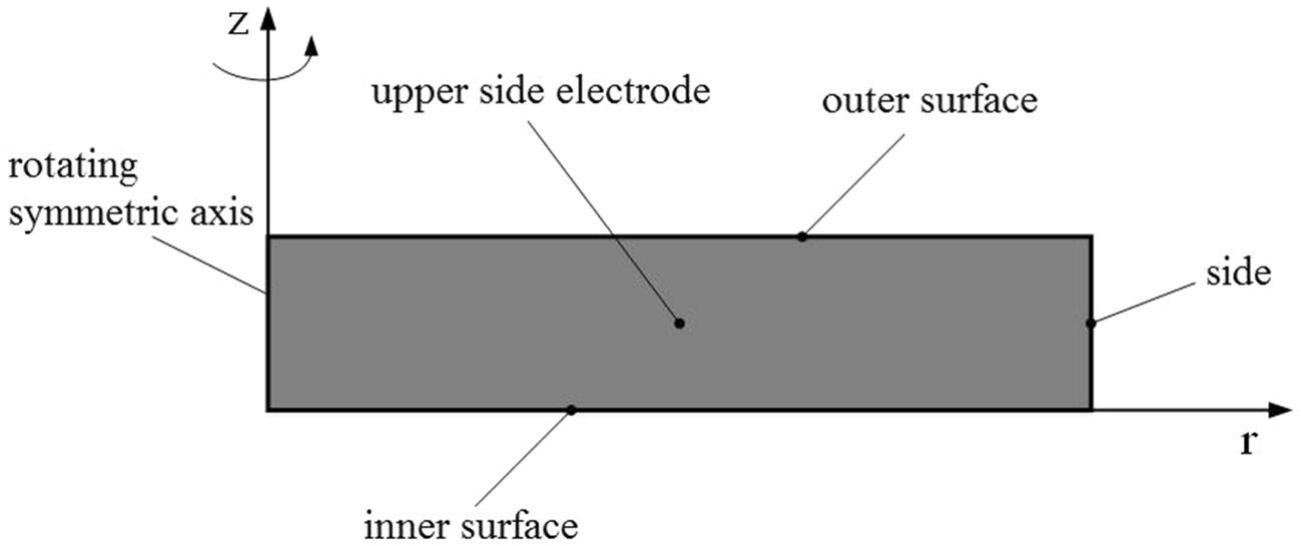
Electrode surface diagram of electrostatic model.

As mentioned above, the charge is concentrated on the surface of the electrode. Taking the upper electrode as an example, the surface of the electrode is analyzed, as shown in Fig. 7. The simulation results show that the charge density on the outer surface is $3.93 \times 10^{{ - 11}}$, the charge density on the side is $3.93 \times 10^{{ - 11}}$, and the charge density on the inner surface is 0.00208. It can be seen that the charge density on the outer surface is two to three orders of magnitude different from that on the side and the inner surface.

According to formula (17), the surface stress tensor of the outer surface is almost negligible, and the stress mainly concentrates on the inner surface and the side. The z-direction stress tensor of the inner surface is 52,222 N/m^2^, while the r-direction stress tensor is $3.93 \times 10^{{ - 11}}$ N/m^2^. It can be seen that the Maxwell stress tensor on the inner surface is extruded inward from the vertical film surface, while the r-direction stress is almost zero, which can be neglected. The stress of the lower electrode is the same.

The simulation results show that the surface stress tensor in the r direction of the upper electrode side is 25,882 N/m^2^, while the surface stress tensor in the lower electrode side is the same, so the surface stress tensor in the r direction of the whole model is 51,764 N/m^2^. The z-direction surface stress tensor of the surface is 1.147 × 10^−8^ N/m^2^, which can be neglected.

It can be concluded that the compressive stress ${\text{p}}_{z}$ of the whole circular film is 52,222 N/m^2^ perpendicular to the plane direction of the film, and the tensile stress ${\text{p}}_{r}$ of the plane direction is 51,764 N/m^2^. The stress calculated by formula (17) is 52,018 N/m^2^. Therefore, the following relationship can be obtained by removing the error of simulation solution:

18 $$ p_{r}  = p_{z}  = \frac{1}{2}\varepsilon _{0} \varepsilon _{r} E^{2} $$

## Conclusions

In this paper, DEG electrostatic model is established, and the distribution of Maxwell stress is determined by theoretical deduction and simulation analysis, and a unified result is obtained. The following conclusions are drawn:

1. According to the theory of electromagnetics and conservation of energy, it is deduced that the film is subjected to the compressive stress in the plane direction and the tensile stress in the plane direction, both of which are $\frac{1}{2}\varepsilon _{0} \varepsilon _{r} E^{2}$.
2. The electrostatic finite element model is established in COMSOL electrostatic module. By applying physical field constraints, the charge density and surface stress tensor on the electrode surface are obtained. Similarly, the compressive stress and the tensile stress in the plane direction of the vertical film are obtained, both of which are $\frac{1}{2}\varepsilon _{0} \varepsilon _{r} E^{2}$.
